# Tuberculosis screening improves preventive therapy uptake (TB SCRIPT) trial among people living with HIV in Uganda: a study protocol of an individual randomized controlled trial

**DOI:** 10.1186/s13063-022-06371-0

**Published:** 2022-05-12

**Authors:** Fred C. Semitala, Lelia H. Chaisson, David W. Dowdy, Derek T. Armstrong, Bishop Opira, Kyomugisha Aman, Moses Kamya, Patrick P. J. Phillips, Christina Yoon

**Affiliations:** 1 Department of Medicine, Makerere University College of Health Sciences, Kampala, Uganda; 2 Infectious Diseases Research Collaboration, Kampala, Uganda; 3 Makerere University Joint AIDS Program, Kampala, Uganda; 4 Division of Infectious Diseases, Department of Medicine, University of Illinois Chicago, Chicago, IL USA; 5 Departments of Epidemiology, International Health, and Medicine, Johns Hopkins University, Baltimore, MD USA; 6 Department of Pathology, Johns Hopkins University, Baltimore, MD USA; 7 Division of Pulmonary and Critical Care Medicine, University of California San Francisco, 1001 Potrero Ave, 5K1, San Francisco, CA 94110 USA

**Keywords:** Tuberculosis, HIV, Screening, C-reactive protein, Tuberculosis preventive therapy, Randomized controlled trial

## Abstract

**Background:**

People living with HIV (PLHIV) have an increased risk of developing active tuberculosis (TB). To reduce the burden of TB among PLHIV, the World Health Organization (WHO) recommends systematic TB screening followed by (1) confirmatory TB testing for all who screen positive and (2) TB preventive therapy (TPT) for all TPT-eligible PLHIV who screen negative. Symptom-based screening remains the standard of care in most high TB burden settings, including Uganda. Despite having high sensitivity for active TB among antiretroviral-naïve PLHIV, symptom screening has poor specificity; as such, many high-risk PLHIV without active TB are not referred for TPT. C-reactive protein (CRP) is a promising alternative strategy for TB screening that has comparable sensitivity and higher specificity than symptom screening, and was endorsed by WHO in 2021. However, the impact of CRP-based TB screening on TB burden for PLHIV remains unclear.

**Methods:**

TB SCRIPT (TB Screening Improves Preventive Therapy Uptake) is a phase 3, multi-center, single-blinded, individual (1:1) randomized controlled trial evaluating the effectiveness of CRP-based TB screening on clinical outcomes of PLHIV. The trial aims to compare the effectiveness of a TB screening strategy based on CRP levels using a point-of-care (POC) assay on 2-year TB incidence and all-cause mortality (composite primary trial endpoint) and prevalent TB case detection and uptake of TPT (intermediate outcomes), relative to symptom-based TB screening (current practice).

**Discussion:**

This study will be critical to improving selection of eligible PLHIV for TPT and helping guide the scale-up and integration of TB screening and TPT activities. This work will enable the field to improve TB screening by removing barriers to TPT initiation among eligible PLHIV, and provide randomized evidence to inform and strengthen WHO guidelines.

**Trial registration:**

ClinicalTrials.gov NCT04557176. Registered on September 21, 2020.

**Supplementary Information:**

The online version contains supplementary material available at 10.1186/s13063-022-06371-0.

## Administrative information

**Table:** 

**Title**	Tuberculosis Screening Improves Preventive Therapy Uptake (TB SCRIPT) trial among people living with HIV in Uganda: a study protocol of an individual randomized controlled trial.
**Trial registration**	Registry: ClinicalTrials.gov Identifier: NCT04557176. Date registered: September 21, 2020.
**Protocol version**	Version 2.1, April 26, 2021.
Funding	NIH/NHLBI: 1R61HL146365.
**Name and contact information for trial sponsor**	University of California, San Francisco. 1001 Potrero Ave, 5 K1. San Francisco, CA 94110, USA.
**Role of the sponsor**	Neither the sponsor nor the funding source has any role in the study design or collection, management, analysis, and interpretation of data. Neither the sponsor nor the funding source had any role in writing the protocol, or the decision to submit the protocol for publication.

## Introduction

### Background and rationale

Tuberculosis (TB) remains the leading killer of people living with HIV (PLHIV) globally, accounting for one-third of all HIV-related deaths [1]. TB preventive therapy (TPT) is highly effective for PLHIV, reducing incident TB by 30–60% [2–7]. When combined with antiretroviral therapy (ART), TPT reduces all-cause mortality by as much as 40% relative to ART alone [3, 6]. Because of the proven benefit of TPT, the World Health Organization (WHO) has declared TPT an essential intervention for all PLHIV without active TB, regardless of latent TB infection (LTBI) status [8, 9]. To support the uptake of TPT among PLHIV, in 2011, the WHO endorsed a 4-part symptom screen (presence of cough, fever, night sweats, and/or weight loss) with high sensitivity and negative predictive value (NPV) to rule out active TB and thus enable the safe initiation of TPT for those who screen-negative [10]. However, this symptom screen has poor specificity among PLHIV, with meta-analyses demonstrating that 50–90% of all PLHIV initiating ART will screen positive for at least one symptom [11–14]. This poses a major barrier to prompt initiation of TPT, as symptom screening alone would require confirmatory testing in half or more of all PLHIV before TPT could be initiated. As such, a higher-specificity screening test to rule out active TB would help facilitate improved TPT initiation for PLHIV.

C-reactive protein (CRP) is a non-specific inflammatory marker whose levels rise in response to infection, including active TB. Prospective studies among individuals with advanced HIV initiating ART have shown CRP to meet the minimum thresholds for sensitivity (≥90%) and specificity (≥70%) established by WHO for an effective TB screening test [15–22], suggesting that CRP could be used to substantially increase TPT uptake among PLHIV and reduce the need for more costly confirmatory testing. Furthermore, CRP can be measured using a low-cost ($2 per test), rapid (results in 3 min), and simple (measured from capillary blood) point-of-care (POC) assay that can be easily implemented in low-resource settings. In response to multiple studies, including an individual patient data meta-analysis, demonstrating its comparable sensitivity and significantly higher specificity compared with symptom screening [15–17, 19, 20, 22], in 2021, the WHO recommended CRP for TB screening in PLHIV [23]. However, CRP is not widely used for TB screening globally, and the impact of CRP-based TB screening on TB case detection, TPT uptake, and TB burden among PLHIV remains unknown.

Here we describe the protocol of TB Screening Improves Preventive Therapy Uptake (TB SCRIPT), a randomized controlled trial conducted in three health centers in Uganda, which was designed to determine if TB screening based on POC CRP concentration improves clinical outcomes (reduced TB incidence and mortality) among outpatients with advanced HIV initiating ART through improved TPT uptake and detection of prevalent TB cases. This work will be critical to both improving selection of eligible PLHIV for TPT and helping guide the scale-up and integration of TB screening and TPT activities in TB-endemic countries.

### Objectives

The primary objective of TB SCRIPT is to compare the impact of POC CRP-based with symptom-based TB screening on 2-year TB disease outcomes. We will randomize participants to either POC CRP-based (intervention) or standard-of-practice symptom-based TB screening (control). All participants who screen positive will undergo confirmatory TB testing, while only participants who screen negative will initiate 12 weekly doses of isoniazid and rifapentine (3HP), a short-course TPT regimen. We will follow all eligible participants for 2 years to assess TB incidence and all-cause mortality. At the final study visit, we will collect sputum to ascertain active TB status from participants who have not been diagnosed with or treated for active TB during follow-up. The primary hypothesis for this objective is that participants randomized to POC CRP-based TB screening will have lower combined 2-year TB incidence and mortality (composite primary trial outcome), relative to participants randomized to symptom-based screening. The second objective is to compare the impact of POC CRP-based with symptom-based TB screening on (1) TPT uptake and (2) prevalent TB case detection, intermediate outcomes on the pathway to the combined primary trial outcome. The primary hypothesis for this objective is that participants randomized to POC CRP-based TB screening will have higher rates of TPT uptake and completion, determined via objective monitoring, and similar rates of prevalent TB case detection relative to participants randomized to symptom-based screening.

### Trial design and study setting

The TB SCRIPT trial is a phase 3, single-blinded, multi-center, randomized superiority trial evaluating the clinical impact of POC CRP-based TB screening on immediate and longer-term outcomes of PLHIV in Uganda. Uganda is among the world’s 30 high HIV/TB burden countries with an HIV prevalence of 6.2% and an annual TB incidence of 253 cases per 100,000 [1, 24]; among all TB cases, HIV prevalence is estimated at 45% [25]. Data from the 2014–2015 national TB prevalence survey estimate that half of all TB cases go undiagnosed each year, and the overall TB burden is greatest in urban areas [24].

**Fig. 1:**
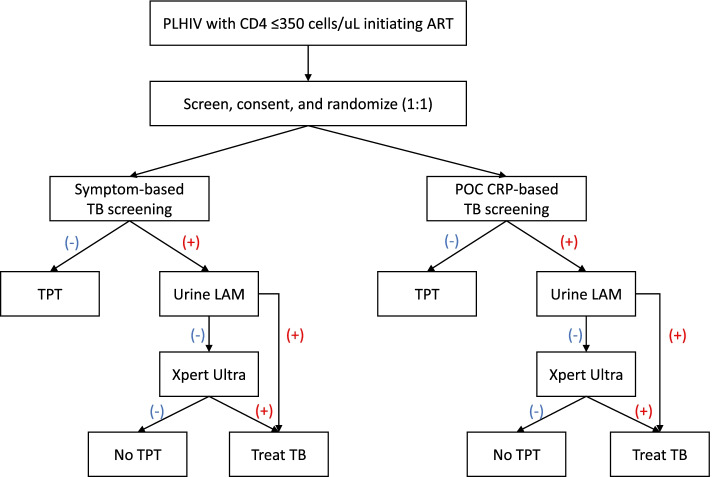
Study enrollment and baseline TB screening. Legend: (+) test-positive; (-) test-negative. Urine LAM-positive participants with risk factors for drug-resistant TB (prior TB treatment, contact with drug-resistant TB case) will undergo sputum Xpert Ultra testing for rapid detection of rifampin-resistance. All participants who test positive for TB (including urine LAM-positive/sputum Xpert Ultra-negative participants) will be referred to routine clinicians for TB treatment initiation

We will randomize 1720 PLHIV presenting for routine ART initiation in three clinics (two public and one non-government/non-profit) in Kampala, Uganda, to either POC CRP-based TB screening (intervention) or symptom-based TB screening (control) with a 1:1 allocation ratio over a 2-year period. After randomization, we will perform baseline TB screening in accordance with the participant’s assigned TB screening strategy. Based on the results of baseline TB screening, we will either collect clinical specimens (urine, sputum) for confirmatory TB testing (urine lipoarabinomannan [LAM], sputum Xpert Ultra MTB/RIF, if screen-positive) or assess 3HP eligibility (if screen-negative, Fig. 1). Supported by study staff, clinic staff will provide all further care and follow-up for HIV, 3HP, and/or active TB, per routine clinic protocols. We will subsequently extract relevant clinical and outcome data from the clinic medical record at regular intervals (months 3, 6, 12, 18, and 24) for 2 years and collect sputum specimens for comprehensive TB testing (sputum Xpert Ultra, culture and drug susceptibility testing [DST]) at the final (24 months) study visit to ascertain 2-year active TB status.

## Methods

### Eligibility criteria

We will screen ART-naïve PLHIV presenting for routine outpatient HIV care for study eligibility. We will enroll adults (age ≥ 18 years) with a confirmed HIV-positive test result and CD4 count ≤350 cells/μL and capacity to provide written (or witnessed verbal, if illiterate) informed consent. We will exclude those who have completed treatment for active pulmonary or extra-pulmonary TB within the past 2 years; completed a full course of any approved TPT regimen within the past year; are actively taking any internationally approved medication for TB treatment (e.g., fluoroquinolones) for any reason, within 2 weeks of study entry; have a prior history of combined ART for HIV treatment for any duration; or who currently reside 25 km outside the enrollment site, have plans to move 25 km outside their enrollment site in the next 2 years, or plan to transfer their HIV care from the enrollment site.

### Informed consent

The study team will obtain written informed consent in English or the local dialect (Luganda). For participants who cannot read or write, we will obtain verbal consent and obtain a thumbprint, and a witness will sign the informed consent document. We will obtain separate written informed consent for collection and storage of biological specimens.

### Interventions

We will compare the effectiveness of two WHO-approved TB screening strategies for PLHIV: POC CRP (intervention) and the WHO 4-part symptom screen (control).

#### Intervention arm: POC CRP-based TB screening

For participants randomized to POC CRP-based TB screening, we will record POC CRP concentrations measured from capillary blood obtained via finger prick using a standard sensitivity POC assay (iCHROMA CRP assay; BodiTech, South Korea). The iCHROMA CRP assay is a US Food and Drug Administration (FDA)-approved, fully quantitative, lateral flow-based fluorescence sandwich immunoassay with a detection range of 2.5–300 mg/L and excellent stability after more than 18 months of storage under the recommended conditions.

Although the recent WHO TB screening guidelines recommend using a cut-point of 5 mg/L to maximize sensitivity of CRP for active TB, we selected a cut-point of 8 mg/L prior to the updated WHO recommendations based on our own previously published research demonstrating a CRP cut-point of 8 mg/L to have 90% sensitivity and 70% specificity for culture-confirmed TB [20, 26, 27]. Participants with POC CRP levels ≥8 mg/L will be classified as screen-positive while those with POC CRP levels < 8 mg/L will be classified as screen-negative.

#### Control arm: symptom-based TB screening

For participants randomized to symptom-based TB screening, we will record the presence or absence of current cough, fever, night sweats, and weight loss in the past 30 days using a standardized script. In accordance with the current WHO and Ugandan TB screening guidelines, participants with ≥1 symptom will be classified as screen-positive and participants with none of the 4 symptoms will be classified as screen-negative [8].

#### Both arms

In both arms, we will use the results of baseline TB screening to guide decisions regarding 3HP initiation and confirmatory TB testing (Fig. 1).

##### Screen-negative participants

We will offer 3HP to all screen-negative participants (POC CRP < 8 mg/L in the intervention arm; absence of all 4 symptoms in the control arm) meeting local 3HP eligibility criteria. 3HP will be initiated no earlier than 2 weeks after ART initiation. Participants initiating 3HP will be managed in accordance with local guidelines and instructed to bring 3HP bottles at every visit during the period of 3HP prescription.

##### Screen-positive participants

We will collect spontaneously voided urine and spot sputum specimens (induced, if necessary) from all screen-positive participants (POC CRP ≥8 mg/L in the intervention arm; presence of ≥1 symptom in the control arm) for urine LAM (Alere Determine TB-LAM) and sputum Xpert Ultra testing. In accordance with current WHO guidelines, sputum Xpert Ultra testing will be performed if (1) urine LAM is negative or (2) urine LAM is positive and the participant has risk factors for drug-resistant TB (prior TB treatment, contact with drug-resistant TB case). Participants who test positive for TB by urine LAM and/or sputum Xpert Ultra will be referred to clinicians for TB treatment initiation. To maximize real-world relevance, sputum will not be submitted for mycobacterial culture because most HIV programs in high burden countries use Xpert (standard cartridge or Ultra) as the confirmatory test for TB. Participants who screen-positive but test negative by urine LAM and sputum Xpert Ultra will not be given 3HP at the time of enrollment (as their risk of active TB remains non-negligible), but will be eligible for TPT initiation at the discretion of treating (non-study) clinicians throughout the follow-up period.

### Participant timeline

After baseline study procedures, routine clinic staff will provide all further care and follow-up for HIV, TPT, and/or active TB treatment. Routine clinical care for all patients initiated on ART includes blood draw for pre-ART labs, HIV viral load at 6 months and as appropriate thereafter, and clinic visits at weeks 2 and 6 and then months 3, 6, 12, 18, and 24 post-ART initiation. Patients initiated on 3HP or TB treatment have additional visits for active TB screening, medication adverse event (AE) monitoring, adherence assessments, and refills, per routine clinic protocols. To capture clinical and outcome data for this trial, we will conduct brief follow-up interviews and extract relevant data from the clinic medical record at regular intervals for 2 years (months 3, 6, 12, 18, and 24). At the final (24-month) study visit, we will collect sputum specimens from all participants who have not been treated for TB during the study for the final active TB assessment. Table 1 describes the schedule of study activities performed by study staff and the schedule of clinic activities performed by routine clinic staff for PLHIV initiating ART.

**Table 1:** Schedule of study activities

Timepoint	Entry	Weeks				Months						
	0	2	4	6	8	3	4	5	6	12	18	24
**Enrollment**												
Eligibility screen	•											
Informed consent	•											
Randomization	•											
**Study activities**												
Baseline TB screening by trial arm	•											
Urine pregnancy testing	•											
Capillary blood β-hCG testing	•											
Banking of serum/plasma and urine	•					•			•	•	•	•
***Screen-negatives***												
TPT eligibility assessment	•											
Liver enzyme testing (if eligible for TPT)	•											
TPT initiation		•										
TPT adherence monitoring				•		•						
***Screen-positives***												
Urine LAM	•											
Xpert Ultra (if LAM-negative or at risk for DR TB)	•											
TB treatment initiation		•										
TB treatment adherence monitoring						•			•			
***Final TB status assessment***												
Xpert Ultra												•
Liquid mycobacterial culture												•
Drug susceptibility testing												•
**Routine clinic activities**												
CD4 count	•											
Blood chemistry	•											
Complete blood count	•											
ART initiation	•											
Routine clinic follow-up visit		•		•		•			•	•	•	•
HIV viral load									•	•	•	•
***Participants initiated on anti-TB therapy***												
AE monitoring		•	•		•	•	•	•	•			
TB treatment adherence monitoring		•	•		•	•	•	•	•			
Medication refills		•	•		•	•	•	•	•			

### Risks to study participants

As this trial is comparing two WHO-endorsed TB screening strategies, the risk of study participation is no greater than routine care. People with prevalent TB may be missed by screening and/or inadvertently initiated on TPT; prevalent TB cases initiated on 3HP are at increased potential risk for developing acquired drug resistance. Missed prevalent TB cases, including all drug-resistant TB cases, will be managed by routine clinicians in accordance with local guidelines. Conversely, PLHIV without active TB may have a false-positive screen and miss the opportunity to receive study-initiated 3HP. The risk of injury to study participants is minimal. In the unlikely event of injury as a result of study participation, standard treatments will be made immediately available at the clinic site and/or Mulago National Referral Hospital; no other form of compensation for injury will be provided.

### Participant retention

To promote retention for participants in both trial arms, we will record participants’ phone numbers and home address at study entry and review/update their contact information at every study visit (with participants’ consent). We will provide phone call reminders of upcoming clinic appointments and reimbursement for transportation expenses for all scheduled study visits.

### Outcomes

The primary trial endpoint is a composite outcome of microbiologically confirmed incident TB and all-cause mortality, measured as time to first diagnosis of incident TB or death from any cause. Microbiologically confirmed incident TB is defined as active TB diagnosed by either culture, one non-trace positive Xpert Ultra result or two trace-positive Xpert Ultra results, or positive urine LAM result from 3 months after randomization to the end of follow-up (at 2 years, or at the time of last contact for participants who are lost to follow-up). To assess active TB status at the end of follow-up, 2 spot sputum specimens will be collected from all participants who have not taken anti-TB treatment during the study at the final (24 months) study visit for sputum Xpert Ultra testing, liquid culture, and drug susceptibility testing (for participants who received any TPT). Secondary outcomes are presented in Table 2.

**Table 2:** Secondary outcomes of the TB SCRIPT trial

Outcome	Numerator	Denominator
**A. TB incidence**		
Number diagnosed with microbiologically confirmed incident TB within 2 years	# diagnosed with incident TB (incident TB = positive non-trace Xpert Ultra result, two trace Xpert Ultra results, or ≥ 1 positive culture result > 3 months after study entry)	None
2-year incidence of microbiologically confirmed TB	# diagnosed with incident TB	# randomized to corresponding study arm - # treated for prevalent TB (prevalent TB = active TB diagnosed ≤3 months of study entry)
Time to microbiologically confirmed incident TB diagnosis	Days from 3 months post-enrollment to incident TB diagnosis (or censoring)	
Incident rate of microbiologically confirmed TB	# diagnosed with incident TB	(# randomized to corresponding study arm - # treated for prevalent TB)/time in person-years
Number diagnosed with drug-resistant incident TB within 2 years	# diagnosed with incident drug-resistant TB (drug-resistant TB = DST-detected resistance to drugs administered for TPT [e.g., isoniazid])	None
2-year incidence of drug-resistant TB among people receiving TPT	# diagnosed with incident drug-resistant TB	# received TPT of any duration
**B. Mortality**		
Number who died from any cause within 2 years	# deaths from any cause	None
2-year all-cause mortality	# deaths from any cause	# randomized to corresponding study arm
Time to death from any cause	Days from enrollment to death from any cause	
All-cause mortality rate	# deaths from any cause	# randomized to corresponding study arm/time in person-years
Number who died from TB within 2 years	# deaths from confirmed TB + # deaths from probable TB (probable TB = review of medical records suggests TB strongly suspected)	None
2-year TB-specific mortality	# deaths from confirmed TB + # deaths from probable TB	# randomized to corresponding study arm
**C. TPT uptake**		
Number screen-negatives prescribed TPT	# screen-negatives prescribed TPT (baseline screen-negatives prescribed TPT ~ study staff-prescribed TPT)	None
Proportion screen-negatives prescribed TPT	# screen-negatives prescribed TPT	# randomized to corresponding study arm
Number screen-positives prescribed TPT	# screen-positives prescribed TPT (baseline screen-positives prescribed TPT ~ routine clinician-prescribed TPT)	None
Proportion screen-positives prescribed TPT	# screen-positives prescribed TPT	# randomized to corresponding study arm
Number initiated on TPT	# screen-negatives prescribed TPT + # screen-positives prescribed TPT	None
Proportion initiated on TPT	# screen-negatives prescribed TPT + # screen-positives prescribed TPT	# randomized to corresponding study arm
Time to TPT initiation	Days from baseline TB screening to initiation of TPT	
Number completing TPT (measured by pill count, self-reported adherence)	# initiated on TPT who completed ≥90% of treatment over prescribed TPT period	None
Proportion completing TPT (measured by pill count, self-reported adherence)	# initiated on TPT who completed ≥90% of treatment over prescribed TPT period	# randomized to corresponding study arm
**D. Prevalent TB diagnosis**		
Number microbiologically confirmed prevalent TB cases detected by the screening test	# screen-positives diagnosed with prevalent TB (diagnosed = a positive urine LAM result, one non-trace positive or two trace-positive Xpert Ultra results ≤3 months of study entry)	
Proportion microbiologically confirmed prevalent TB cases detected by the screening test	# screen-positives diagnosed with prevalent TB	# randomized to corresponding study arm
Number microbiologically confirmed prevalent TB cases missed by the screening test	# screen-negatives diagnosed with prevalent TB	None
Proportion microbiologically confirmed prevalent TB cases missed by the screening test	# screen-negatives diagnosed with prevalent TB	# randomized to corresponding study arm
Number diagnosed with microbiologically confirmed prevalent TB	# screen-positives diagnosed with prevalent TB + # screen-negatives diagnosed with prevalent TB	None
Microbiologically confirmed TB prevalence	# screen-positives diagnosed with prevalent TB + # screen-negatives diagnosed with prevalent TB	# randomized to corresponding study arm
**E. Prevalent TB treatment**		
Number treated for prevalent TB	# treated (treated = initiated on TB treatment ≤3 months of study entry regardless of Xpert Ultra result)	None
Proportion treated for prevalent TB	# treated	# randomized to corresponding study arm
Number with microbiologically confirmed prevalent TB completing treatment	# diagnosed AND treated who complete treatment (complete treatment = clinic TB treatment records confirm treatment completion)	None
Proportion with microbiologically confirmed prevalent TB completing treatment	# diagnosed AND treated who complete treatment	# diagnosed
Time to treatment of microbiologically confirmed prevalent TB	Days from prevalent TB diagnosis to initiation of TB treatment	

### Sample size

The sample size calculations are primarily based on the primary objective (which requires a larger sample size). We expect 2-year mortality of 5.2% and 2-year TB incidence of 7.3% in the control arm [4, 28]. Assuming a two-sided alpha of 0.05 and 12% lost to follow-up, a sample size of 860 participants per arm (1720 total) will provide 80% power to detect a relative reduction of 35% in the 2-year combined outcome (from 12.5% [*n* = 95] to 8.1% [*n* = 62]). This sample size will also provide 85% and 90% power if 2-year mortality is 6.8% and 8.8%, respectively (Table 3). This sample size also gives 80% power to detect a smaller 29% relative reduction if the 2-year combined TB incidence and mortality in the control arm is as high as 17.5% with a 10% loss to follow-up. We will analyze the primary outcome as time-to-event and therefore expect this study power assuming binary outcomes will be a lower bound for study power.

**Table 3:** Power calculations (power to detect 35% difference in the combined 2-year TB incidence and mortality outcome when 2-year TB incidence is 7.3%)

2-year mortality		Power
POC CRP-based screening	Symptom-based screening	
3.4%	5.2%	0.80
4.4%	6.8%	0.85
5.1%	7.9%	0.88
5.7%	8.8%	0.90

For the secondary objective, based on published estimates of specificity from similar settings, we estimate no more than 15% of all patients in the control arm will initiate 3HP (and 13.5%, or 90% of all control patients initiating 3HP, will complete 3HP) [16, 17, 20]. This sample size will give more than 90% power to show improvements in the proportion of all patients initiating 3HP by an absolute difference of 6% of more, which is much less than what is considered clinically important; we anticipate 55% of all patients in the POC CRP will initiate 3HP (and 55% of all patients will complete 3HP).

### Random allocation

Randomization lists were generated in R using Chen’s design with a biased coin and maximum tolerated imbalance, stratified by site [29]. Within each site, sequentially numbered pre-printed forms with each participant arm allocation were inserted into opaque envelopes, which were sealed and sequentially numbered. Randomization is implemented by the trained study staff member opening the next unopened envelope in sequence revealing the randomization arm assigned to the participant, only after all eligibility criteria have been assessed and the participant has been found to be eligible.

### Blinding

TB SCRIPT is a single-blinded trial. We will not be blinded to intervention allocation, as we will perform baseline TB screening and subsequent procedures based on the results of screening (e.g., 3HP initiation for eligible screen-negative participants). Participants and routine clinic staff will be blinded, as will the study principal investigators (except for the trial statistician). To facilitate blinding, all participants will undergo a finger prick and have capillary blood measured for CRP (intervention) or beta-human chorionic gonadotropin (β-hCG; control, regardless of gender). POC CRP and β-hCG assays will be provided by BodiTech, Med Inc. and measured using the iCHROMA Reader which provides analysis of more than 30 unique markers, including CRP and β-hCG. There are no procedures for unblinding, as clinicians are free to order TB testing, initiate TB treatment, or initiate TPT for study participants at any time.

### Data collection

#### Medical record review

All participating clinics will use standardized electronic case report forms for initial and follow-up visits. At the baseline visit, we will administer a standardized questionnaire to verify relevant information in the medical record including demographic data, TB and other opportunistic infection history, current medications, and routine lab measurements. At follow-up visits, we will review the medical record to extract relevant interval medical history (e.g., TPT-related adverse events, incident TB diagnosis), new medications (e.g., clinician-initiated active TB treatment or TPT), and routine lab measurements (e.g., HIV viral load) using a standardized form.

#### TPT and TB treatment adherence monitoring

For participants receiving 3HP, we will assess adherence by performing pill counts and patient-reported adherence. In addition, we will review the medical record and record the number of 3HP refill visits completed. For participants receiving active TB treatment, we will review clinic TB treatment records and record the number of treatment months recorded as complete.

#### Prevalent TB status assessment

For all participants who screen-positive by their assigned TB screening strategy, we will collect urine and 2 spot sputum specimens for urine LAM (Alere Determine TB-LAM) and sputum Xpert Ultra testing. All urine LAM-negative participants and urine LAM-positive participants with risk factors for drug-resistant TB (history of prior TB treatment, contact with a known drug-resistant TB case) will have sputum tested by Xpert Ultra for rapid detection of rifampin resistance. All participants with a positive urine LAM and/or sputum Xpert Ultra result (including trace-positives) will be referred to routine clinic staff for initiation of anti-TB treatment. Additionally, for participants with trace-positive Xpert Ultra results, we will perform repeat Xpert Ultra testing on the second spot sputum specimen. Participants with a non-trace-positive Xpert Ultra result or two trace-positive Xpert Ultra results will be classified as having active prevalent TB.

#### Final TB status assessment

For all participants who have not taken anti-TB treatment during follow-up, we will collect 2 spot sputum specimens at the final (24 months) study visit for sputum Xpert Ultra testing; repeat Xpert Ultra testing, if the initial test is trace-positive; and liquid culture and drug susceptibility testing (DST). Speciation testing will be performed on all positive cultures to confirm the presence of *M. tuberculosis* complex using Ziehl-Neelsen smear microscopy for detection of acid-fast bacilli and SD MPT64 (Standard Diagnostics Bioline TB Ag MPT64, South Korea) for rapid *M. tuberculosis* antigen detection. DST will include susceptibility testing to all first-line TB medications including isoniazid and rifamycin-based drugs. Participants will be classified as having incident TB if at least one of the following criteria are met: at least one sputum culture positive for *M. tuberculosis* complex, one non-trace-positive Xpert Ultra result, or two trace-positive Xpert Ultra results.

### Data management

Data collection, entry, management, and quality assurance are conducted at each enrollment site. However, all standard operating procedures for data collection and management are identical across the enrollment sites. We have developed standardized REDCap data collection forms and implemented procedures for quality control. Data collection forms provide clear instructions to guide data entry and contain pre-programmed skip patterns, real-time range checks, and internal logic to minimize missing data and facilitate cleaner data at capture. Study data will be entered directly (in real-time) into REDCap using password-protected laptop computers or tablets. Data entered in Uganda while offline will be uploaded to a secure University of California San Francisco server on a daily basis. All data will undergo weekly checks for completeness, consistency, accuracy, and range criteria. Queries performed on the database will be reviewed, verified, and corrected every 2 weeks.

### Statistical methods

#### Primary and secondary outcomes

The primary trial outcome is a 2-year composite endpoint of microbiologically confirmed incident TB and all-cause mortality, measured as time to first diagnosis of incident TB or death from any cause. The primary analysis will be by intention-to-treat (ITT), including all participants randomized without regard to the results of baseline TB screening, and we will use a two-sided alpha (0.05 significance) in assessing the effect of the intervention on the primary outcome. Participants with prevalent TB will be included in the analysis to preserve the randomized groups (the ITT principle) but excluded from the outcome of incident TB because subsequent disease is more likely to represent relapse or failure (and these participants will not routinely receive TPT). Time from randomization to first event (diagnosis of incident TB or death from any cause) will be analyzed using the log rank test and Cox proportional hazards models, adjusted for the study site. If data are missing or a patient is lost to follow-up, a patient’s data will be censored in the analysis at the date of last known contact. We will also use the Mantel-Haenszel method to determine standardized incidence rates and incidence rate differences. Balance at baseline on all known and suspected prognostic factors will be assessed, and if important imbalances are found (e.g., differences in median baseline CD4 count), a sensitivity analysis will be conducted using a Cox model to adjust for these factors. We will also assess the impact of clinician-initiated co-interventions (e.g., clinician-initiated TPT, clinician-initiated TB treatment) on the primary outcome. We will conduct a sensitivity analysis in which these participants are censored as soon as they are initiated on TPT or TB treatment by non-study clinicians. Standard checks for variation by enrollment site will also be planned. Secondary outcomes for the primary objective include the individual primary endpoints (TB incidence and mortality analyzed separately) and 2-year isoniazid-resistant TB incidence and will be analyzed using a similar approach; there will be no adjustment for multiplicity since these are pre-specified secondary outcomes.

To evaluate intermediate outcomes on the pathway to the composite primary trial outcome, we will determine the proportion of randomized participants initiating TPT (prescribed TPT), the proportion of randomized participants completing TPT (≥90% TPT pills taken), the proportion of randomized participants diagnosed with prevalent TB, and the proportion of randomized participants with prevalent TB completing TB treatment (by clinic record review). The primary analysis will be by ITT, with the outcome of initiating any TPT based on actual events, irrespective of whether the algorithm was followed. Participants eligible for 3HP based on the results of baseline screening (i.e., study-initiated TPT) may decline to initiate 3HP after randomization despite being prescribed 3HP; these participants will be counted as not having started TPT. Participants who were initiated on TPT during study follow-up (i.e., clinician-initiated TPT for participants who were classified as ineligible for 3HP based on the results of baseline screening) will be counted as having started TPT; 6 months of daily isoniazid (IPT) is currently the most common TPT regimen available for routine clinical use. We will compare the proportion of PLHIV initiating any TPT (study- and clinician-initiated TPT) and the proportion of PLHIV completing TPT (the denominator will be the total number of participants in the study arm), calculating the risk difference and 95% confidence intervals (CI) using standard methods, adjusted for study site using Cochran-Mantel-Haenzsel weights. If respective data on an outcome is missing or a patient is lost to follow-up, it will be assumed that the patient did not initiate nor complete TPT (depending on what data are available). Our working hypothesis is that POC CRP-based TB screening will increase the proportion of PLHIV initiating and completing TPT by at least 40%, relative to symptom-based screening. We will compare the proportion of PLHIV with screen-positive prevalent TB and the proportion of PLHIV with screen-positive prevalent TB completing TB treatment, calculating the risk difference and 95% CIs using standard methods, adjusted for study site using Cochran-Mantel-Haenszel weights. Our working hypothesis is that the proportion of PLHIV with screen-positive prevalent TB will be similar in each arm, and we expect the upper bound of the CI of the difference will be no more than 5%. We consider this margin of 5% sufficiently small to conclude equivalence.

We will repeat the primary analysis in the following pre-specified sub-groups: by age, gender, enrollment site, and CD4 strata (< 50, 50–199, ≥200 cells/μL). Safety data related to missed diagnoses of prevalent TB, acquired drug-resistant TB, and TPT-related serious adverse events will be tabulated by study arm.

### Plans to give access to the full protocol, participant-level data, and statistical code

The full protocol, participant-level dataset, and statistical code will be made available upon request if agreed upon by the principal investigators.

### Oversight and monitoring

The study will be overseen by an Administrative Core, chaired by the principal investigator and Uganda site principal investigator. In addition, a Clinical Trial Core chaired by two principal investigators will be responsible for maintaining ethics approvals, databases, and study monitoring. An Economic Modeling Core chaired by a principal investigator will be responsible for overseeing all economic and costing analyses.

The study team meets bi-weekly via conference calls to monitor the progress of the study, participant enrollment, and retention. Bi-weekly conference calls include a review of trial outcomes, as well as factors external to the study (e.g., scientific developments, COVID-19) that could impact the safety of study participants and/or study activities. Additional monthly conference calls separately review all adverse events and protocol deviations.

Day-to-day data and safety monitoring is the responsibility of the principal investigators and will follow guidelines established by IRBs regarding reportable adverse events (AE). Reportable AEs are reported to the IRBs within 10 days of study awareness. Reportable AEs will be attributed to the study intervention by the principal investigator with review by the physicians/co-investigators according to the following categories: definite, probably, possible, unlikely, and unrelated. A bi-annual and annual report of all reportable AEs will be presented to the Data Monitoring Committee (DMC) and National Heart, Lung and Blood and Institute, respectively. In addition, an endpoint adjudication committee will meet annually to review all deaths and determine causes of death.

### Data monitoring committee

An independent DMC that includes experts in TB diagnostics and the analytical and regulatory aspects of clinical trials met by conference call before enrollment and will meet at 25%, 50%, 75%, and 100% enrollment and when 50% of patients have completed follow-up and when 100% have completed follow up to review safety data from the trial. They are charged with evaluating the quality of trial administration, monitoring safety issues, and providing guidance on scientific, methodological, and ethical issues. Specifically, the DMC will review administrative reports prepared by the Data Manager for each meeting of the DMC. This report will describe study progress, including the following: (1) accrual, (2) demographics, (3) subject status, (4) TB prevalence, (5) proportion of patients initiating TPT, (6) number and type of serious AEs (SAE), (7) cumulative TB incidence (including drug-resistant TB), and (8) cumulative all-cause mortality. Tables by arm will be prepared for review in the closed sessions of the DMC meetings; the DMC will have access to screening assignments. Differences in the composite primary trial outcome (2-year TB incidence and 2-year mortality) between trial arms are unlikely to modify our study protocol because of the long duration of follow-up (2 years), relative to recruitment. However, differences in missed diagnoses of prevalent TB (primary risk and safety endpoint), measured as the proportion of microbiologically confirmed prevalent TB cases missed by screening, will be carefully reviewed and the decision to continue enrollment after each meeting will be made by the DMC. Although unlikely, the DMC should inform the PIs if, in their view, the results of an interim analysis are likely to convince a broad range of clinicians (including those supporting the trial and the general clinical community) that, on balance, one trial arm is clearly indicated or contraindicated for all participants or a particular category of participant. Further details of stopping guidelines and roles and responsibilities of members are included in the DMC charter.

### Frequency and plans for auditing trial conduct

Internal monitoring will occur annually, in accordance with the trial’s Comprehensive Quality Management Plan (CQMP).

## Discussion

TB remains a leading infectious cause of death globally and is the leading cause of death for PLHIV [1]. Diagnosing and treating TB in PLHIV through intensified case finding, and preventing TB through TPT among TB-negative PLHIV, are key for global TB control; however, symptom-based screening — the most common strategy for TB screening in high burden settings — has poor specificity, resulting in underuse of TPT. CRP has higher specificity than symptom screening in PLHIV and was endorsed by WHO in 2021 [23]; however, the long-term clinical and public health impact of CRP-based screening remains unclear. The TB SCRIPT trial will be critical to improving selection of eligible PLHIV for TPT and helping guide the scale-up and integration of TB screening and TPT activities. This work will help improve TB screening globally by removing barriers to TPT initiation among eligible PLHIV and provide randomized evidence to inform and strengthen WHO guidelines.

### Trial status

Protocol version 2.1, April 26, 2021. Recruitment began on November 16, 2020, and is currently ongoing. As of October 20, 2021, 558 individuals were screened for study eligibility and 491 were enrolled. Recruitment completion is estimated in December 2022. Trial registration: ClinicalTrials.gov, NCT04557176 (https://clinicaltrials.gov/ct2/show/NCT04557176).

## Supplementary Information

**Additional file 1:** Biological specimens

## Data Availability

All authors will have full access to the final trial dataset. The full protocol, participant-level dataset, and statistical code will be made available upon request if agreed upon by the Principal Investigators.

## Abbreviations

3HP: 3-Months weekly isoniazid, rifapentine, and pyridoxine
β-hCG: Beta-human chorionic gonadotropin
AE: Adverse event
ART: Antiretroviral therapy
CI: Confidence interval
CRP: C-reactive protein
CQMP: Comprehensive Quality Management Plan
DMC: Data Monitoring Committee
DST: Drug susceptibility testing
FDA: Food and Drug Administration
HIV: Human immunodeficiency virus
ITT: Intention-to-treat
LAM: Lipoarabinomannan
LTBI: Latent tuberculosis infection
NPV: Negative predictive value
PLHIV: People living with HIV
POC: Point-of-care
SAE: Serious adverse event
TB: Tuberculosis
TB SCRIPT: TB Screening Improves Preventive Therapy Uptake
TPT: Tuberculosis preventive therapy
US: United States
WHO: World Health Organization
