# Supplementary material for: Tuberculosis screening improves preventive therapy uptake (TB SCRIPT) trial among people living with HIV in Uganda: a study protocol of an individual randomized controlled trial
Source: Trials. 2022 May 12;23:399. doi: 10.1186/s13063-022-06371-0 (PMC9096738; doi:10.1186/s13063-022-06371-0)
Supplement: Supplementary file 1 — Additional file 1: Biological specimens [file 13063_2022_6371_MOESM1_ESM.docx]

**Biological specimens**

We will obtain informed consent from participants to obtain and store biological specimens for future research. For consenting participants, we will collect blood (25mL) and urine (30mL) at the baseline and final study visits. Specimens will be stored at Makerere University-UCSF Laboratory and UCSF. Samples will be labeled with the same unique study ID for each participant that renders the data anonymous to persons outside the study. Samples will be stored indefinitely.
